# Targeted genetic analysis unveils novel associations between ACE I/D and APO T158C polymorphisms with D-dimer levels in severe COVID-19 patients with pulmonary embolism

**DOI:** 10.1007/s11239-022-02728-z

**Published:** 2022-11-13

**Authors:** Giuseppe Fiorentino, Giuditta Benincasa, Antonietta Coppola, Monica Franzese, Anna Annunziata, Ornella Affinito, Mario Viglietti, Claudio Napoli

**Affiliations:** 1 Department of Intensive Care, A.O.R.N. Ospedali dei Colli, Naples, Italy; 2 Department of Advanced Medical and Surgical Sciences (DAMSS), University of Campania “Luigi Vanvitelli”, Naples, Italy; 3 IRCCS SDN, Naples, Italy

**Keywords:** Genetic testing, Cardiovascular risk factors, Severe COVID-19, Pulmonary embolism, D-Dimer

## Abstract

Only a percentage of COVID-19 patients develop thrombotic complications. We hypothesized that genetic profiles may explain part of the inter-individual differences. Our goal was to evaluate the genotypic distribution of targeted DNA polymorphisms in COVID-19 patients complicated (PE+) or not (PE−) by pulmonary embolism. We designed a retrospective observational study enrolling N = 94 consecutive patients suffering severe COVID-19 with pulmonary embolism (PE+, N = 47) or not (PE−, N = 47) during hospitalization. A panel of N = 13 prothrombotic DNA polymorphisms (FV R506Q and H1299R, FII G20210A, MTHFR C677T and A1298C, CBS 844ins68, PAI-1 4G/5G, GPIIIa HPA-1 a/b, ACE I/D, AGT T9543C, ATR-1 A1166C, FGB − 455G > A, FXIII103G > T) and N = 2 lipid metabolism-related DNA polymorphisms (APOE T 112C and T158C) were investigated using Reverse Dot Blot technique. Then, we investigated possible associations between genotypic subclasses and demographic, clinical, and laboratory parameters including age, obesity, smoking, pro-inflammatory cytokines, drug therapy, and biomarkers of thrombotic risk such as D-dimer (DD). We found that 58.7% of PE+ had homozygous mutant D/D genotype at ACE I/D locus vs. PE− (40.4%) and 87% of PE+ had homozygous mutant C/C genotype at APOE T158C locus vs. PE− (68.1%). In PE+ group, DD levels were significantly higher in D/D and I/D genotypes at ACE I/D locus (P = 0.00066 and P = 0.00023, respectively) and in C/C and T/C genotypes at APOE T158C locus (P = 1.6e−06 and P = 0.0012, respectively) than PE− group. For the first time, we showed significant associations between higher DD levels and ACE I/D and APOE T158C polymorphisms in PE+ vs. PE− patients suggesting potential useful biomarkers of poor clinical outcome.

**Supplementary Information:**

The online version contains supplementary material available at 10.1007/s11239-022-02728-z.

## Highlights

- Only a fraction of COVID-19 patients develop thrombotic complications.
- Genetic background may affect the individual susceptibility to develop pulmonary embolism in COVID-19 patients.
- ACE I/D and APO T158C polymorphisms associated with higher levels of D-dimer in COVID-19 patients with pulmonary embolism.
- ACE I/D and APO T158C polymorphisms may explain part of the individual susceptibility to poor clinical outcome.

## Introduction

Complex genetic and epigenetic molecular circuits can underlie the strong propensity to thrombotic events in patients with coronavirus disease 2019 (COVID-19), especially in those patients who are affected by the severe form of disease; however, the molecular components of the individual risk predisposing to thrombosis have not been fully established yet [1–5]. COVID-19 has a wide spectrum of possible clinical features ranging from asymptomatic disease to severe interstitial pneumoniae and acute respiratory distress syndrome (ARDS) [2, 3].

Retrospective studies showed that increased levels of D-dimer (DD), procalcitonin, and C Reactive Protein (CRP) may be prognostic biomarkers useful to predict the onset of thrombotic complications [6]. Moreover, the incidence of thrombotic events (about 31%) [7] was associated with increased morbidity and mortality in critically ill patients [8]. At molecular level, the thrombotic manifestations of severe COVID-19 patients seem to be consequences of direct SARS-CoV-2 cytotoxic effects, endothelialitis, dysregulated immune response, platelet aggregation, and complement and coagulation cascade activation [1, 8]. But none of these molecular pathways is sufficient to explain why some COVID-19 patients develop thrombotic manifestations and other do not.

Genetic risk factors related to a pre-existing prothrombotic state seem to play a key role in determining the individual susceptibility both to SARS-CoV-2 infection and clinical course of disease [9, 10]. We conducted a retrospective, single-centre, observational study to evaluate the genotypic distribution of N = 15 polymorphisms involved in thrombotic risk and lipid metabolism in COVID-19 patients who developed acute pulmonary embolism (PE+) as compared to those who did not develop acute pulmonary embolism (PE−).

## Materials and methods

### Study population

In this retrospective, single-center, observational study, we enrolled 94 consecutive hospitalized patients (22 F and 72 M) suffering severe COVID-19 who were admitted to the Sub-Intensive Care Unit of A.O.R.N. Ospedali dei Colli, Cotugno Hospital, Naples (Italy) between 13 January 2021 and 2 May 2021. The inclusion criteria were: (1) respiratory rate 30 breaths/min; (2) arterial oxygen saturation 93% at rest; (3) PaO2/FiO_2_ < 300 mmHg; (4) mechanical ventilation; (5) patients with and without pulmonary embolism. Exclusion criteria were: (1) pediatric age, (2) patients with no genetic testing, and (3) patients missing clinical or laboratory data of interest. Severe COVID-19 patients were stratified in two groups based on the presence of pulmonary embolism (PE+, N = 47) or the absence of pulmonary embolism (PE−, N = 47). Upon admission, we retrieved anamnestic and anthropometric parameters [age, gender, body mass index (BMI), smoking habit], presence of comorbidities [systemic arterial hypertension, type 2 diabetes (T2D), coronary heart disease (CHD), and chronic obstructive pulmonary disease (COPD)], drug therapy, including angiotensin-converting enzyme inhibitors (ACEi) or sartans, and routine laboratory parameters, such as CRP, prothrombin time (PT), activated partial thromboplastin time (aPTT), prothrombin time and international normalized ratio (PT-INR), DD, fibrinogen, interleukin (IL)-2 receptor (IL2R), and IL-6.

### Ethics statement

This study was approved by the local ethics committee (AOC-0017432-2020). Each study participant provided written informed consent prior to study enrolment, collection of samples and subsequent genetic analysis. This study was conducted according to the principles and guidelines expressed in the Declaration of Helsinki.

### Blood collection and genetic testing

Upon admission, from each study participant we collected about 5 mL of whole blood from a peripheral vein in EDTA tubes. Then, the molecular analysis was performed by our Hospital Central Laboratory by using a Reverse Dot Blot (RDB) kit by Nuclear Laser Medicine (NLM, version 2020.10.19, CVD-14cod. AC084, Milan). We determined the individual genotypes at 15 loci including FV R506Q and FV H1299R, FII G20210A, MTHFR C677T and MTHFR A1298C, CBS 844ins68, PAI-1 4G/5G, GPIIIa HPA-1 a/b, ACE I/D, AGT T9543C, AGTR-1 A1166C, FGB-455G > A, and FXIII103G > T, APOE T112C, and APOE T158C (Table 1).

**Table 1:** Distribution of genotypic frequencies in PE− vs. PE+ COVID-19 patients

Polymorphism	PE−	PE+	P
FV R506Q, n (%)			0.43
G/G (homozygous wild type)	45 (95.7)	42 (91.3)	
G/A (heterozygous)	2 (4.3)	4 (8.7)	
AA (homozygous mutant)	0 (0.0)	0 (0.0)	
FV H1299R, n (%)			0.66
A/A (homozygous wild type)	41 (87.2)	38 (82.6)	
A/G (heterozygous)	6 (12.8)	7 (15.2)	
G/G (homozygous mutant)	0 (0.0)	1 (2.2)	
FII, n (%)			0.20
G/G (homozygous wild type)	46 (97.9)	42 (91.3)	
G/A (heterozygous)	1 (2.1)	4 (8.7)	
A/A (homozygous mutant)	0 (0.0)	0 (0.0)	
MTHFR C677T, n (%)			0.77
C/C (homozygous wild type)	15 (31.9)	15 (32.6)	
C/T (heterozygous)	20 (42.6)	22 (47.8)	
T/T (homozygous mutant)	12 (25.5)	9 (19.6)	
MTHFR A1298C, n (%)			0.06
A/A (homozygous wild type)	22 (46.8)	15 (32.6)	
A/C (heterozygous)	24 (51.1)	24 (52.2)	
C/C (homozygous mutant)	1 (2.1)	7 (15.2)	
CBS 844ins68, n (%)			**0.03**
D/D (homozygous wild type)	38 (80.9)	44 (95.7)	
I/D (heterozygous)	9 (19.1)	2 (4.3)	
I/I (homozygous mutant)	0 (0.0)	0 (0.0)	
PAI-1-675 (4G/5G), n (%)			0.67
5G5G (homozygous wild type)	10 (21.3)	10 (21.7)	
4G5G (heterozygous)	30 (63.8)	26 (56.5)	
4G4G (homozygous mutant)	7 (14.9)	10 (21.7)	
HPA1 T1565C GP IIIa, n (%)			1.00
T/T (homozygous wild type)	29 (61.7)	30 (65.2)	
T/C (heterozygous)	16 (34.0)	15 (32.6)	
C/C (heterozygous)	2 (4.3)	1 (2.2)	
ACE I/D, n (%)			**0.04**
I/I (homozygous wild type)	5 (10.6)	8 (17.4)	
I/D (heterozygous)	23 (48.9)	11 (23.9)	
D/D (homozygous mutant)	19 (40.4)	27 (58.7)	
APOE T112C, n (%)			0.36
T/T (homozygous wild type)	43 (93.6)	38 (82.6)	
T/C (heterozygous)	3 (6.4)	7 (15.2)	
C/C (homozygous mutant)	1 (2.1)	1 (2.2)	
APOE T158C, n (%)			**0.02**
T/T (homozygous wild type)	0 (0.0)	1 (2.2)	
T/C (heterozygous)	15 (31.9)	5 (10.9)	
C/C (homozygous mutant)	32 (68.1)	40 (87.0)	
AGT T9543C, n (%)			0.82
T/T (homozygous wild type)	12 (25.5)	10 (21.7)	
T/C (heterozygous)	25 (53.2)	23 (50.0)	
C/C (homozygous mutant)	10 (21.3)	13 (28.3)	
AGTR-1 A1166C, n (%)			0.63
A/A (homozygous wild type)	28 (59.6)	26 (56.5)	
A/C (heterozygous)	14 (29.8)	17 (37.0)	
C/C (homozygous mutant)	5 (10.6)	3 (6.5)	
FBG -455, n (%)			0.14
G/G (homozygous wild type)	21 (44.7)	30 (65.2)	
G/A (heterozygous)	22 (46.8)	13 (28.3)	
A/A (homozygous mutant)	4 (8.5)	3 (6.5)	
F XIII, n (%)			0.12
G/G (homozygous wild type)	29 (61.7)	34 (73.9)	
G/T (heterozygous)	14 (29.8)	12 (26.1)	
T/T (homozygous mutant)	4 (8.5)	0 (0.0)	

Statistically significant p-values (p < 0.05) are highlighted in bold

### Statistical analysis

A statistical post-hoc power analysis was performed to compute power values for given sample sizes, effect size, and alpha level. By selecting one-tailed Wilcoxon-Mann–Whitney test with 2 groups (PE+ vs. PE−), with a sample size of 47 for both groups, a medium effect size (f = 0.5) and an alpha level of 0.05, the power resulted around 0.76. Thus, our sample size was more than adequate for the main objective of this study and for possible subgroup analysis. G*Power software version 3.1 was used to compute the power analysis.

Statistical analysis was performed using R Core Team (version 4.0. 0). Continuous variables were expressed as mean and standard deviation (SD). Data distribution was tested for normality through the Shapiro–Wilk test. Unpaired Student’s t-test or Wilcoxon rank-sum test, as required, were performed for comparison between two groups. Categorical variables were expressed as a percentage and were compared using the Chi-Square test or the Fisher’s exact test. A P < 0.05 was considered significant. Bonferroni’s correction was used for multiple hypothesis correction, if necessary. A Spearman’s correlation analysis was run to investigate whether there was an association among variables in two groups separately. A Spearman’s ρ with P ≤ 0 0.05 was set as threshold to identify an agreement between variables. For the multivariate analysis, a logistic regression model was performed to evaluate risk factors associated with pulmonary embolism, considering thrombotic and cardiovascular parameters.

## Results

### Clinical characteristics of study population

Clinical characteristics of COVID-19 patients are summarized in Table 2. A total of 94 severe COVID-19 patients were included in the study with a mean age of 58.98 ± 12.33 years and 60.94 ± 12.14 years for PE− and PE+ groups, respectively. In both groups there was a prevalence of males (72.3% and 80.9% for PE− and PE+, respectively). Besides, we found a higher percentage of patients falling in the “non-smoking” class both in PE− (57.4%) and PE+ (51.1%) as compared to patients falling in the “smoking” class (PE−, 29.8% and PE+, 17%) and patients falling in the “former-smoking class” (PE−, 12.8% and PE+, 31%). In addition, a higher percentage of patients were not obese (PE−, 74.5% and PE+, 68.1%) or affected by T2D (PE−, 76.6% and PE+, 63.8%) but showed hypertension (PE−, 63.8% and PE+, 59.6%). Only a small percentage of patients was affected by CHD (PE−, 19.1% and PE+, 14.9%) or COPD (PE−, 6.4% and PE+, 6.4%). Regarding pharmacological treatment, we found that a higher percentage of patients did not use ACEi (PE−, 57.4% and PE+, 63.8%) or sartans (PE−, 70.2% and PE+ 61.7%). No statistical differences were detected for all these parameters in PE+ vs. PE− patients. As expected, higher DD levels were found in PE+ vs. PE− groups with a statistical significance (P = 5.4e−09, Wilcoxon rank-sum test after Bonferroni correction) (Table 3 and Supplementary Fig. 1). Otherwise, differences in CRP, PT, aPTT, PT-INR, fibrinogen, IL2R, and IL6 were not statistically significant between the two groups (Table 3). Multivariate logistic regression analysis revealed that D-Dimer was an independent risk factor significantly associated with PE (p-value = 0.015). As reported in Supplementary Table 1, none of the other clinical variables (age, BMI, IL2R, IL6, CRP, PT, aPTT, PT-INR and fibrinogen) reached statistical significance.

**Table 2:** Baseline clinical characteristics of COVID-19 patients

Parameter	PE−	PE+	P
Gender, n (%)			0.33
F	13 (27.7)	9 (19.1)	
M	34 (72.3)	38 (80.9)	
Obesity, n (%)			0.49
No	35 (74.5)	32 (68.1)	
Yes	12 (25.5)	15 (31.9)	
Smoking, n (%)			0.06
Former	6 (12.8)	15 (31.9)	
No	27 (57.4)	24 (51.1)	
Yes	14 (29.8)	8 (17.0)	
T2D, n (%)			0.18
No	36 (76.6)	30 (63.8)	
Yes	11 (23.4)	17 (36.2)	
Hypertension, n (%)			0.67
No	17 (36.2)	19 (40.4)	
Yes	30 (63.8)	28 (59.6)	
CHD, n (%)			0.58
No	38 (80.9)	40 (85.1)	
Yes	9 (19.1)	7 (14.9)	
COPD, n (%)			1.00
No	44 (93.6)	44 (93.6)	
Yes	3 (6.4)	3 (6.4)	
ACEi, n (%)			0.53
No	27 (57.4)	30 (63.8)	
Yes	20 (42.6)	17 (36.2)	
SARTANS, n (%)			0.38
No	33 (70.2)	29 (61.7)	
Yes	14 (29.8)	18 (38.3)	

*ACEi* angiotensin-converting enzyme inhibitors, *CHD* coronary heart disease, *COPD* chronic obstructive pulmonary disease, *F* female, *M* male, *T2D* type 2 diabetes

**Table 3:** Characteristics and laboratory parameters of COVID-19 patients

Clinical parameters	PE+			PE−			p-value
	n	mean	sd	n	mean	sd	
Age	47	58.9	12.3	47	60.9	12.1	0.2
BMI	47	28.4	4.6	47	28.6	4.7	0.8
CRP	47	7.9	7.5	47	9.6	8.7	0.3
IL2R	47	1059.1	619.3	47	1073.8	461.5	0.5
IL6	47	148.9	319.1	47	273.3	780.1	0.2
PT	47	81.5	13.2	47	81.1	17.1	1
aPTT	47	32.3	5.1	47	31.4	4.4	0.4
PT-INR	47	0.7	0.5	47	0.8	0.5	0.1
D-DIMER	47	408.5	802.7	47	2999.6	6015.5	5.41E-09
Fibrinogen	47	508.4	218.9	47	462.4	201.6	0.2

### Genotyping

We evaluated a panel of 15 polymorphisms already known to be associated with increased thrombotic risk and lipid metabolism dysfunction (Table 1). We found that the genotypic distribution at ACE I/D (P = 0.04), CBS 844ins68 (P = 0.03), and APOE T158C (P = 0.02) loci significantly discriminated PE+ vs. PE− (Table 1). According to genotypic subclass distribution, we found that ACE I/D polymorphism showed: (1) D/D genotype (homozygous mutant) in 58.7% of PE+ vs. 40.4% of PE−; (2) I/D genotype (heterozygous) in 23.9% of PE+ vs. 48.9% of PE−; and (3) I/I genotype (homozygous wild type) in 17.4% PE+ vs. 10.6% of PE−; CBS 844ins68 polymorphism showed: (1) D/D genotype (homozygous wild type) in 95.7% of PE+ vs. 80.9% of PE−, (2) I/D genotype (heterozygous) in 4.3% of PE+ vs. 19.1% of PE−; and APOE T158C polymorphism showed: (1) C/C genotype (homozygous mutant) in 87% of PE+ vs. 68.1% of PE−, (2) T/C genotype (heterozygous) in 10.9% of PE+ vs. 31.9% of PE−, and (3) T/T genotype (homozygous wild-type) in 2.2% of PE+ vs. 0% of PE−. In contrast, FV R506Q and H1299R, FII G20210A, MTHFR C677T and A1298C, PAI-1 4G/5G, GPIIIa HPA-1 a/b, AGT T9543C, ATR-1 A1166C, FGB-455G > A, FXIII103G > T, and APOE T 112C did not show a significant different distribution between PE+ and PE− patients (Table 1).

### Associations between ACE I/D polymorphism and clinical parameters

Based on our interest on ACE I/D polymorphism and its documented association with the severity of COVID-19 in Europe [11–15], we investigated potential associations between genotypic subclasses and clinical and laboratory parameters. As showed in Supplementary Fig. 2, in PE+ patients we observed: (1) a moderate positive correlation between IL2-R and fibrinogen (FB) (ρ = 0.44, P = 0.02) and a significant negative correlation between PT and PT-INR (ρ = 0.4, P = 0.045) for D/D genotype; (2) a significant positive correlation between BMI and PT (ρ = 0.67, P = 0.03) for I/D genotype; and (3) a significant positive correlation between CRP and DD levels (ρ = 0.7, P = 0.004) and between IL6 levels and aPTT (ρ = 0.88, P = 0.004), as well as a significant negative correlation between IL6 levels and PT-INR (ρ =  − 0.71, P = 0.048) for I/I genotype. In PE− patients (Supplementary Fig. 3), we observed: (1) a significant positive correlation between IL2R and PT-INR (ρ = 0.64, P = 0.003), a moderate positive correlation between PT-INR and FB (ρ = 0.5, P = 0.03) and a significant negative correlation between PT and age (ρ = − 0.63, P = 0.004) for D/D genotype; (2) a moderate positive correlation between CRP and IL6 levels (ρ = 0.46, P = 0.03), age and BMI (ρ = 0.43, P = 0.04), PT-INR and IL2R (ρ = 0. 41, P = 0.049) and PT-INR and DD levels (ρ = 0.45, P = 0.03) as well as a moderate negative correlation between CRP and age (ρ = − 0.43, P = 0.03), PT and aPTT (ρ = − 0.47, P = 0.02) and PT and PT-INR (ρ = − 0.45, P = 0.03) for I/D genotype; and (3) a significant positive correlation between CRP, IL6 and DD levels (ρ = 1, P <  < 0.01; ρ = 0.9, P = 0.04) as well as between IL6 and DD levels (ρ = 0.9, P = 0.04) for I/I genotype.

### Association of DD levels with ACE I/D and APOE T158C polymorphisms

**Fig. 1:**
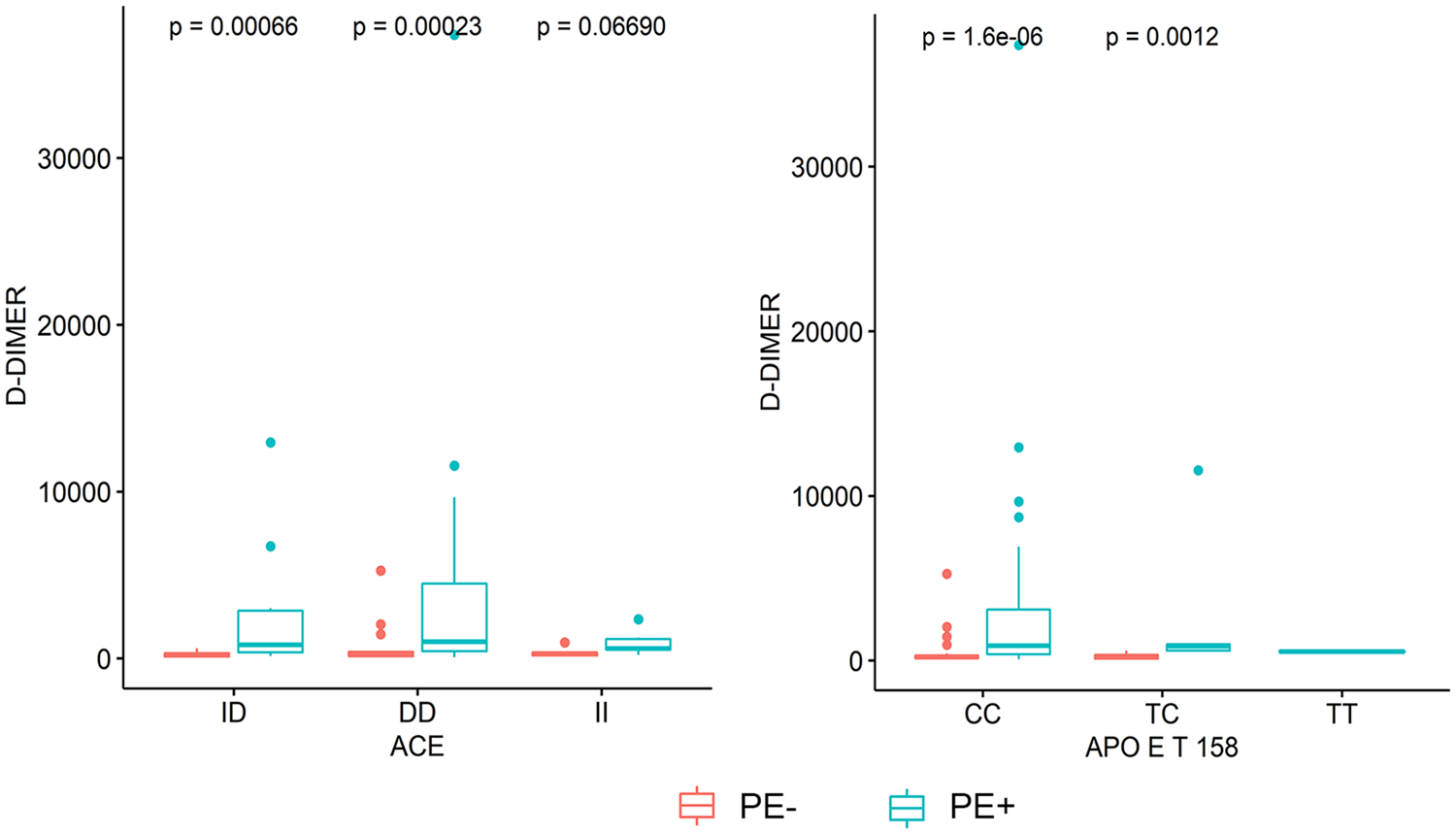
Association of DD plasma levels with ACE I/D and APOE T158C genotypic subclasses in severe COVID-19 patients. In PE+ patients, DD levels were significantly higher in carriers of D/D and I/D genotypes at ACE I/D locus (P = 0.00066 and P = 0.00023, respectively) (on the right panel) and in carriers of C/C and T/C genotypes at APOE T158C locus (P = 1.6e−06 and P = 0.0012, respectively) (on the left panel)

We investigated the distribution of DD levels according to the ACE I/D and APOE T158C (Fig. 1, on the left panel) genotypic subclasses. First, in PE+ patients DD levels were significantly higher in carriers of D/D and I/D genotypes at ACE I/D locus (respectively, P = 0.00066 and P = 0.00023) (Fig. 1, on the right panel) and in carriers of C/C and T/C genotypes at APOE T158C locus (respectively P = 1.6e−06 and P = 0.0012) as compared to PE− patients. No significant difference was observed in DD levels between the three genotypic subclasses at ACE I/D and APOE T158C loci both in PE+ and PE− patients (Supplementary Fig. 4).

## Discussion

Our major findings were as follow: (1) genotypic differences at ACE I/D (P = 0.04), CBS 844ins68 (P = 0.03), and APOE T158C (P = 0.02) loci discriminated significantly PE+ vs. PE−; (2) a higher percentage of homozygous mutant ACE D/D and APOE C/C genotypes was found in PE+ vs. PE− patients; (3) in PE+ patients, DD levels were significantly higher in carriers of D/D and I/D genotypes at ACE I/D locus and in carriers of C/C and T/C genotypes at APOE T158C locus.

Previous data showed a higher prevalence of ACE D/D genotype in severe COVID-19 patients as compared to those with less severe disease [12, 13, 16]. At genetic level, the ACE I/D polymorphism is distinguished by either an insertion (I) or deletion (D) of 287 base pairs (Alu repeat segment) in the intron 16. In the general population, the carriers of the mutant D allele had higher ACE protein plasma and tissue levels (ACE levels in D/D carriers were approximately twice that in I/I carriers) as well as elevated levels of the vasopressor angiotensin II and reduced half-life of the vasodilator bradykinin as compared to carriers of the I allele [17, 18]. Beyond the systemic hypertension, ACE D/D genotype was significantly associated to cardiometabolic diseases, such as obesity and T2D, which are known risk factors for COVID-19 [19]. Therefore, it is plausible that ACE I/D polymorphism may play a key role in COVID-19 patients who are susceptible to develop severe lung injury or ARDS. Also, the racial difference of ACE I/D polymorphism is well established. In European populations (Italy, Spain, and France), there is a higher frequency of D allele up to 82% to 87% [20] than Eastern Asian populations (Chinese, Korean, Taiwanese, and Japanese) which have a higher frequency of ACE I allele (33% to 51%) [21]. Globally, it seems that the racial variance of ACE I/D genotype coincides with the differences of outcome; in fact, populations with higher D allele frequency (e.g., Italian) experienced higher fatality (https://www.worldometers.info/coronavirus/#countries). *ACE* gene shows potent vasoconstrictive effects, attenuation of fibrinolysis, and platelet activation and aggregation, and ACE I/D polymorphism represents a susceptibility risk biomarker for thrombosis. In particular, ACE D/D genotype was associated to thromboembolic manifestations in patients affected by other diseases with no pre-existing risk factors and traditional thrombophilia-related polymorphisms [22], increased venous thromboembolism risk in patients with a thrombogenic condition [23, 24], and hypercoagulability and endothelial damage in hypertensive patients [25]. At global level, our data corroborated previous evidence about the increased DD plasma levels in severe COVID-19 patients with thrombotic complications [6, 26]. Screening the three genotypic subclasses, we found for the first time a statistically significant association between increased plasma DD levels and carriers both of D/D and I/D genotypes in PE+ vs. PE− patients. Despite there is need to validate molecular mechanisms underlying this association, we hypothesized that the unbalance of ACE/ACE2 levels characterizing the D/D and I/D genotypes might induce apoptotic processes which target the endothelial cells of the vascular structure leading to coagulopathy and thus increased DD levels in COVID-19 patients.

The APOE locus has been associated with increased vulnerability to severe COVID-19 and mortality, especially for the APOE4 homozygous genotype (ε4/ε4) [27], which is the strongest genetic risk factor for sporadic Alzheimer’s disease. The APOE ε4 allele is characterized by the presence of a C at the APOE T112C locus and a C at the APOE T158C [28]. Our analysis showed that PE+ patients had a higher percentage of homozygous mutant C/C genotype at APOE T158C locus vs. PE− patients, even if we did not find statistically significant difference for the APOE T 112C locus. Also, DD levels were significantly higher in carriers of C/C and T/C genotypes at APOE T158C locus.

During pandemic, inherited thrombophilia has been associated with severe COVID-19 [29–31]. However, there is not a rigorous consensus regarding the genes which are truly involved in inherited thrombophilia. For example, the MTHFR polymorphism is considered as “benign” without a validated link to thrombosis thus questioning its inclusion in the current thrombophilia laboratory test panels [32]. In our study, we did not find statistical differences for MTHFR C677T polymorphism in PE+ vs. PE−, as well as for the other polymorphisms included in the panel. Considering the MTHFR C677T polymorphism, the prevalence of the homozygous TT genotype was 25.5% and 19.6% in PE− vs. PE+ patients (Table 1). These distributions are quite similar to known global levels of the MTHFR C677T polymorphism, for which the prevalence of the homozygous TT genotype was about 26% and 20% in Campania and Sicily (South Italy), respectively [33].

Nevertheless, our small sample size did not allow a secure finding but limited our observation to a hypothesis generating study.

The unique exception was the CBS 844ins68 polymorphism. Surprisingly, D/D homozygous wild-type genotype was most represented in PE+ (95.7%) vs. PE− (80.9%) patients. This might be due to the relatively small number of patients recruited in the study. As in oncology and cardiovascular diseases, the study of genetic regulation should be coupled with the investigation of epigenetic regulation of prothrombotic genes in severe COVID-19 patients and their possible involvement in immune reactivity [5, 34–42].

Our study has some limitations. First, this is a retrospective study. Second, we focused on COVID-19 patients with and without PE, with no control group (non-COVID-19 patients and/or healthy subjects) limiting the attribution of results to a specific association with COVID-19. Second, PE+ patients showed a higher prevalence of homozygous mutant ACE D/D genotype and APOE C/C genotype vs. PE− patients; but homozygous mutant ACE D/D genotype and APOE C/C genotype were also found in 40.4% and 68.1% of PE− patients (Table 1). This suggests a quite high absolute prevalence of the homozygous mutant polymorphisms in these genes. This somehow limits the predictivity of the mutant genotype for PE+.

## Conclusions

Our data documented a differential distribution of homozygous mutant ACE D/D and APOE C/C genotypes between PE+ and PE− patients. Higher DD plasma levels significantly associated with carriers of D/D and I/D genotypes at ACE I/D locus as well as with carriers of C/C and T/C genotypes at APOE T158C locus in PE+ vs. PE− patients. These associations and the potential pathogenic role of these polymorphisms needs to be further evaluated in larger prospective studies.

## Supplementary Information

Below is the link to the electronic supplementary material.

Supplementary file1 (DOCX 558 kb)

## Data Availability

The data that support the findings of this study are available from the corresponding author, [GB], upon reasonable request.

## Abbreviations

ACE: Angiotensin-converting enzyme
ACEi: Angiotensin-converting enzyme inhibitors
AGT: Angiotensinogen
AGTR-1: Angiotensin II type 1 receptor
APOE: Apolipoprotein
aPTT: Activated partial thromboplastin time
ARDS: Acute respiratory distress syndrome
BMI: Body mass index
CBS: Cystathionine β-synthase
CHD: Coronary artery disease
COPD: Chronic obstructive pulmonary disease
COVID-19: Coronavirus disease 2019
CRP: C reactive protein
DD: D-Dimer
FGB: Fibrinogen β
FII: Factor II
FiO2: Fractional inspired oxygen
FV: Factor V
FXIII: Factor XIII
GPIIIa: Platelet glyciprotein IIIA
IL: Interleukin
MTHFR: Methylenetetrahydrofolate reductase
PAI-1: Plasminogen activator inhibitor-1
PaO2: Arterial oxygen partial pressure
CRP: C reactive protein
PE: Pulmonary embolism
PT: Prothrombin time PT-INR
PT-INR: Protrombin time-international normalized ratio
RDB: Reverse dot blot
T2D: Type 2 diabetes
