# Supplementary material for: Targeted genetic analysis unveils novel associations between ACE I/D and APO T158C polymorphisms with D-dimer levels in severe COVID-19 patients with pulmonary embolism
Source: J Thromb Thrombolysis. 2022 Nov 13;55(1):51–9. doi: 10.1007/s11239-022-02728-z (PMC9660132; doi:10.1007/s11239-022-02728-z)
Supplement: Supplementary file 1 — Supplementary file1 (DOCX 558 kb) [file 11239_2022_2728_MOESM1_ESM.docx]

**Supplementary Figure 1**


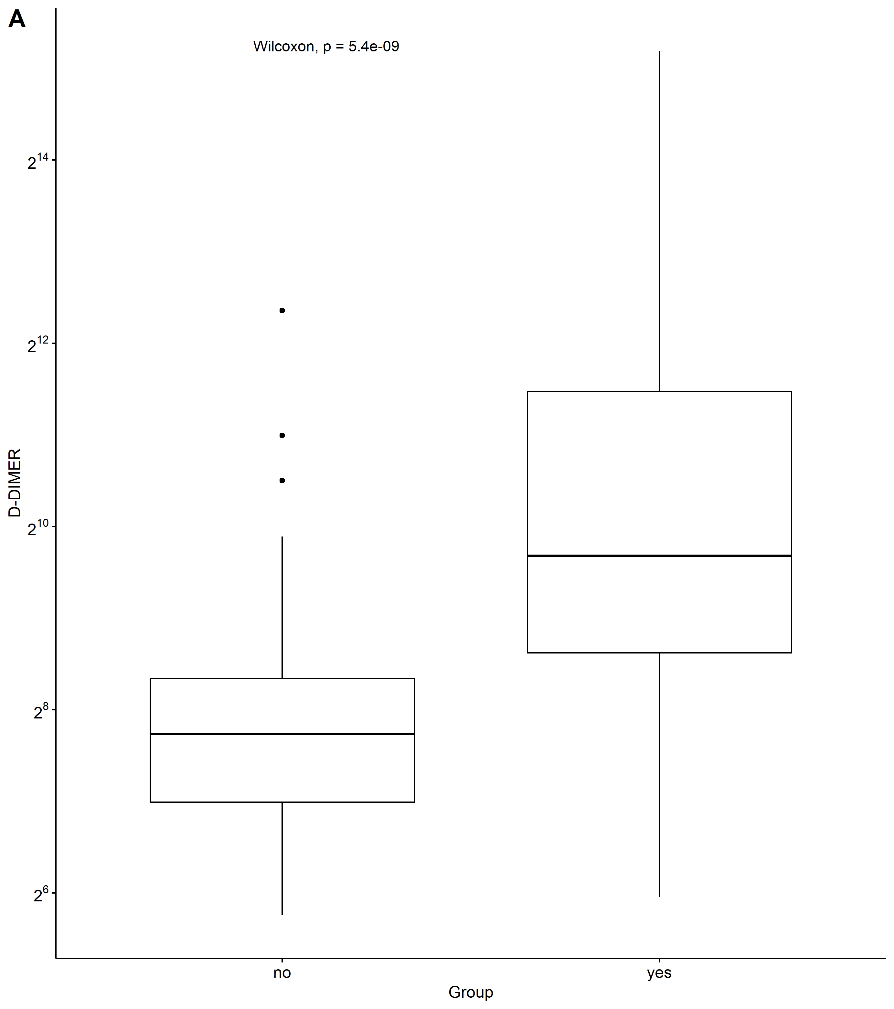


**Plasma levels of DD in COVID-19 patients.** Higher levels of plasma DD significantly distinguished PE+ *vs.* PE- patients(p=5.4e- 09, Wilcoxon rank-sum test).

**Supplementary Figure 2**


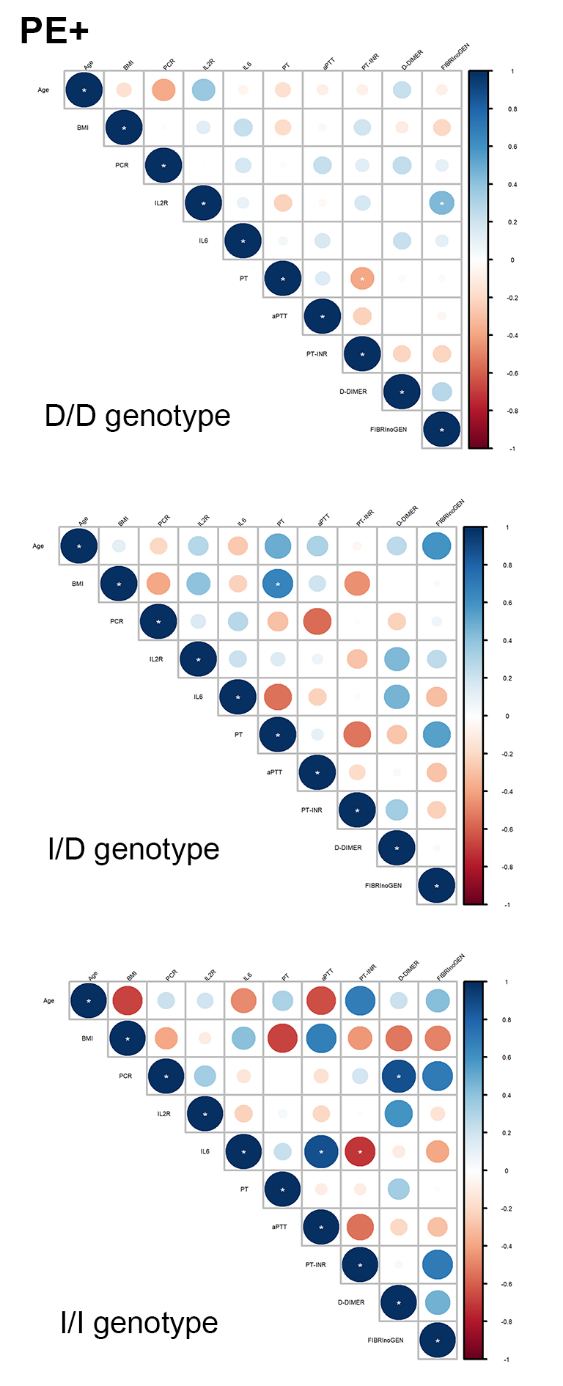


**Association of I/D genotypic subclasses and clinical parameters in PE+ patients.** Correlograms show the Spearman’s correlation between clinical and laboratory parameters in PE+ patients according to the three genotypic subclasses at ACE I/D locus. The asterisk (*) indicates significant correlations (P ≤ 0.05).

**Supplementary Figure 3**

**
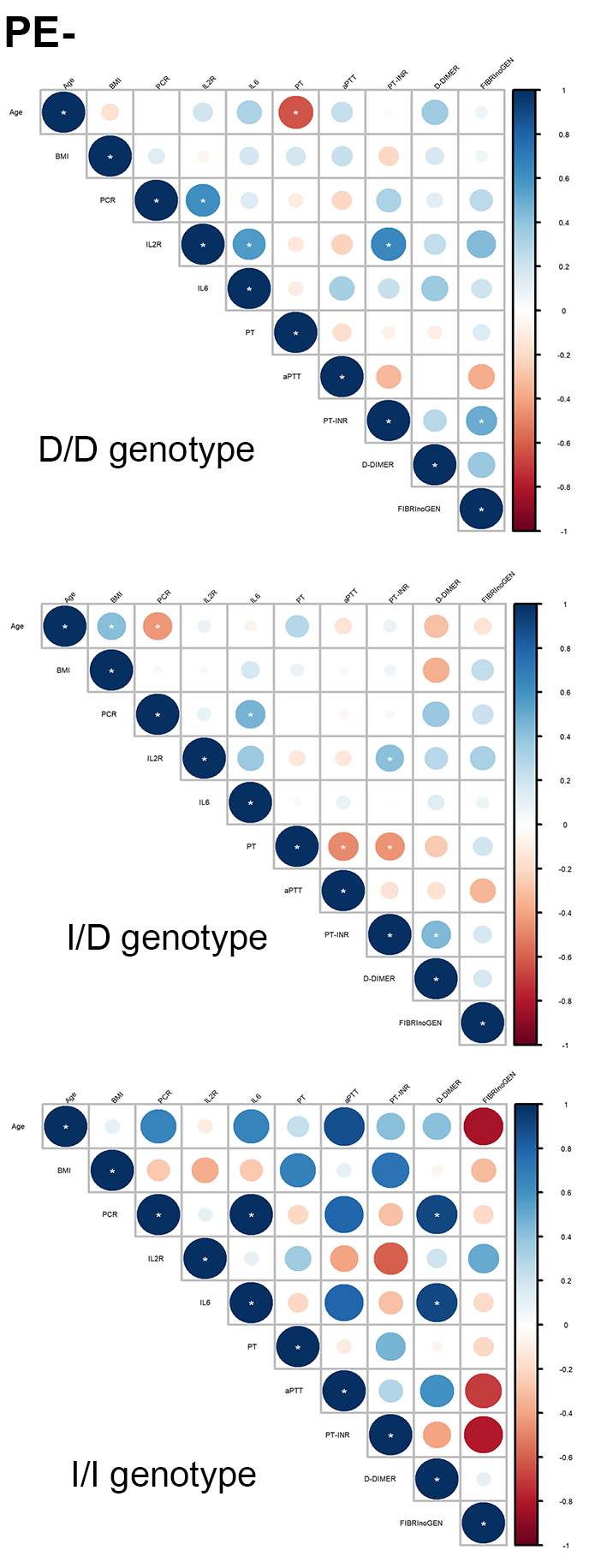
**

**Association of I/D genotypic subclasses and clinical parameters in PE- patients.** Correlograms show the Spearman’s correlation between clinical and laboratory parameters in PE+ patients according to the three genotypic subclasses at ACE I/D locus. The asterisk (*) indicates significant correlations (P≤0.05).

**Supplementary Figure 4**


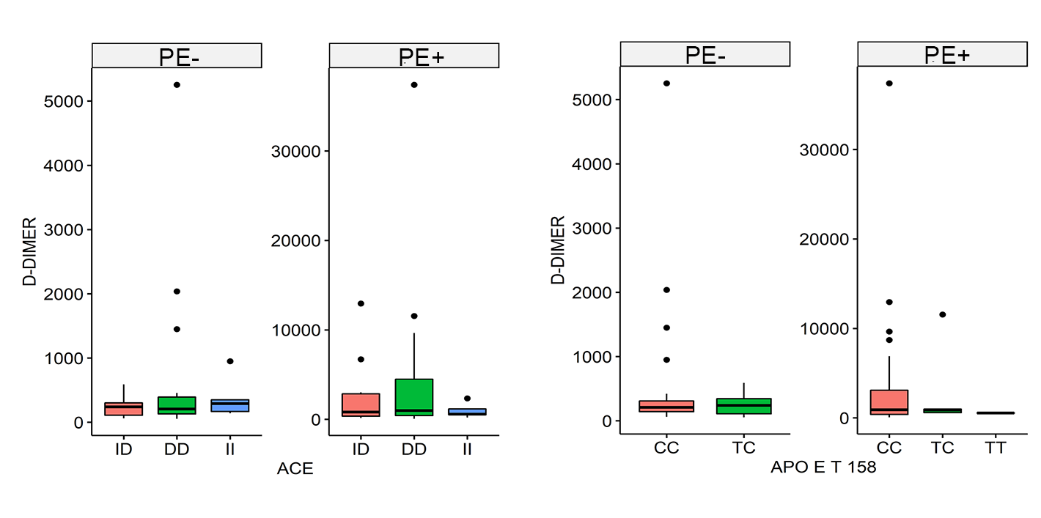


**Plasma levels of DD and genotypic distribution analysis.**No significant difference was observed in DD plasma levels between the three genotypic subclasses at both ACE I/D and APOE T158C loci in PE+ vs. PE- patients.

**Supplementary Table 1**

|  | **Estimate** | **Std. Error** | ***p*value** |
| --- | --- | --- | --- |
| **AGE** | 8.006e-03 | 2.133e-02 | 0.7075 |
| **BMI** | 2.355e-02 | 5.602e-02 | 0.6743 |
| **D-DIMER** | 1.049e-03 | 4.319e-04 | 0.0151 * |
| **IL2R** | -7.232e-05 | 5.398e-04 | 0.8934 |
| **IL6** | 7.538e-04 | 8.219e-04 | 0.3591 |
| **CRP** | 1.483e-02 | 3.326e-02 | 0.6557 |
| **PT** | 5.223e-03 | 1.742e-02 | 0.7643 |
| **aPTT** | -2.984e-02 | 6.182e-02 | 0.6293 |
| **PT-INR** | 8.726e-01 | 5.621e-01 | 0.1206 |
| **FIBRINOGEN** | -2.334e-03 | 1.322e-03 | 0.0774 |

**Multivariate regression analysis for thrombotic and cardiovascular risk factors in PE- vs. PE+ patients.**
